# Utilization and Associated Factors of Insecticide Treated Bed Net among Pregnant Women Attending Antenatal Clinic of Addis Zemen Hospital, North-Western Ethiopia: An Institutional Based Study

**DOI:** 10.1155/2018/3647184

**Published:** 2018-12-24

**Authors:** Azeb Ewinetu Yitayew, Habtamu Demelash Enyew, Yitayal Ayalew Goshu

**Affiliations:** ^1^Department of Midwifery, College of Health Sciences, Debre Tabor University, Debre Tabor, Ethiopia; ^2^Department of Social and Public Health, College of Health Sciences, Debre Tabor University, Debre Tabor, Ethiopia

## Abstract

**Introduction:**

Insecticide treated bed net (ITN) is one type of cost-effective vector control approach for the prevention of malaria. It has to be treated with insecticide and needs ongoing treatment with chemicals. Malaria infcetion during pregnancy is a amajor health problem in Ethiopia. Little is known about the utilization of ITN by pregnant women in the study area. This study was aimed to assess utilization and associated factors of insecticide-treated nets among pregnant women in Adis Zemen Hospital.

**Methods:**

This hospital based cross-sectional study was conducted in Adis Zemen from May 1 to 30, 2018, among 226 pregnant mothers. After obtaining informed consent, data were collected using a pretested structured questionnaire via face to face interview. To reach the study unit, a systematic random sampling technique was used. The collected data were entered, cleaned, checked using Epi data version 3.1, and finally analyzed using SPSS version 20. Binary and multivariable logistic regressions were computed to identify significantly associated variables at 95% confidence interval.

**Result:**

A total of 226 pregnant mothers attending antenatal clinics participated in making the response rate 100%. Among a total 226 subjects, 160(70.8%) of mothers had good utilization of insecticide bet net. Mothers who had an educational status of college and above were 2.8 times more likely to utilize insecticide-treated bed net than mothers who could not read and write (AOR; 2. 8: CI; 1.9, 6.5). Mothers whose age was >30 were 70% times less likely utilized insecticide-treated bed net than mothers whose age was 30 and less (AOR;.3: CI;.2,.6).

**Conclusion and Recommendation:**

Utilization of insecticide-treated bed net by pregnant women is low in the study area. The participants' age, educational status, household monthly income, and husband educational status were significantly associated with utilization of insecticide-treated bed net. Different stakeholders shall give a special attention to awareness creation on advantageous of insecticide bed net.

## 1. Introduction

Insecticide-treated bed net (ITN) is one type of cost-effective vector control approach for the prevention of malaria and it has to be treated with insecticide and needs ongoing treatment. It implies that using ITN is very helpful way in the prevention of malaria transmission in highly endemic areas [1].

Malaria is an infectious disease which can be transmitted from person to person through biting of female mosquitoes [2, 3]. Malaria remains a preventable cause of serious death and illness worldwide, including Ethiopia. In 2016, in estimation 216 million cases of malaria occurred worldwide. From this report, 90% of cases were reported from the African Region with 80% of the report from sub-Saharan Africa. According to WHO 2017, there were a total of 445, 000 deaths due to malaria in 2016 of which 90% of deaths were from the African region [4].

Despite malaria affects all ages and sexes, its infection, severity, recurrence, complication, and malaria related death is very common in pregnant women and children less than five years of age due to low immunity during pregnancy and under 5 age [4, 5].

Different analytical studies reveal that infection of malaria during pregnancies has bad pregnancy outcomes like miscarriage, maternal anemia, stillbirth, intrauterine growth restriction, low birth weight, neonatal sepsis, and prematurity [6–8]. Prematurity and neonatal sepsis are the 1st and 2nd leading causes of neonatal mortality in Ethiopia as evidenced by Mini-Ethiopian Demographic Health Survey, 2014 [9]. This indicates that malaria infection during pregnancy is a major health problem of newborn and mother.

Malaria during pregnancy is the major common problem worldwide and it is the common indirect cause of maternal mortality. The burden of malaria during pregnancy, including its complication is high in African countries, especially sub-Saharan countries including Ethiopia. In Ethiopia nearly about 75% of its total area is malarious and about 65% of its population is at risk of developing malaria infection [9–11]. Studies indicate that in estimation 25 million pregnancies are at risk of developing malaria in sub-Saharan Africa every year. With this high number of pregnancies which are at risk, the consequence of malaria is very high for both the mother and the child in terms of morbidity and mortality [12–14].

In Ethiopia, the prevalence of malaria during pregnancy varies from 6.1% to 10.4%, which is a public health problem. But, this terrible problem can be eliminated or reduced by appropriate utilization of insecticide treated nets for all pregnant women [15–17]. Using of appropriate ITNs is considered as a key in reducing the adverse effects of malaria during pregnancy among the vulnerable populations. The effective use of effective ITN is shown to reduce malaria transmission by 90% and miscarriages and stillbirths by 33% [4, 7, 8].

In order to control this high burden of malaria during pregnancy, the federal ministry of health has distributed 29.6 million long-lasting insecticidal nets (LLINs) which represent 60% of the total population [18].

Even if the magnitude of malaria during pregnancy is high and there are different strategies to decrease the risk of malaria for vulnerable groups, many articles revealed that the use of ITNs by the pregnant woman in sub-Saharan countries including Ethiopia is very low [15–26]. And this low utilization of ITNs by pregnant women is affected by education status, occupation, residence, ownership of television or radio, religion, ethnicity, age, and family monthly income [27–37].

A cross-sectional study was conducted in Raya Zebo district, Ethiopia, to assess utilization of ITN and its associated factors among pregnant woman of the predominantly rural population and the finding revealed that 22.2% of pregnant women reported sleeping under ITN the night before the survey [25]. According to another cross-sectional study which was done in Shahsogo wereda, Ethiopia, among pregnant women, 15.8% of participants owned at least one ITN. From those pregnant women who owned ITN, 7.5% of participants had good practice of ITN utilization [26].

Moreover, little is known about the utilization of ITN by pregnant women in the study area. By considering this gap, this study was aimed to assess utilization and associated factors of insecticide-treated nets among pregnant woman in the Adis Zemen Hospital which, is helpful in guiding policymakers and concerned bodies to give emphasis about utilization of ITNs by the pregnant woman.

## 2. Methods

### 2.1. Setting

A hospital-based cross-sectional study was conducted from May1-30, 2018, in Adis Zemen hospital. Addis Zemen is an administrative town of Libo Kemkem Wereda which is located 656 kilometers away from Addis Ababa and 90 kilometers far from Bahirdar (the capital city of Amhara Regional State). The town is divided into three kebelles (the smallest unit of the woreda) and has an estimated total population of 42, 983 consisting of 21, 749 (50.6%) women.

According to 2015 Adis Zemen town health statistics report, the estimated total population is 42, 983 of whom 21, 234 (49.4%) are men and 21, 749, 609 (50.6%) are women. The total number of women in the reproductive age group (15-49 years) is 14, 248 which accounts for 33.1% of the total town population. The town has one district hospital, one health center, and two private clinics. Adis Zemen Hospital was established in 2015 with a total of 91 staffs and, currently, the hospital has a total of 236 staff [31].

### 2.2. Participants

All pregnant women who attended antenatal clinics of the Adis Zemen Hospital were the source of population and all pregnant women who attended antenatal clinics of the Adis Zemen Hospital during the study period were the study population.

### 2.3. Sample Size Determination and Sampling Procedure

The sample size was calculated using single population proportion formula by assuming 15.8% population proportion of ITNs utilization [28] with 95% confidence interval, the marginal error of 5% (0.05), and 10% nonresponse rate. The sampled 226 pregnant women were selected by systematic sampling technique. The sampling interval was determined by dividing the total number of client flows within one month by sample size. According to the hospital report, the average cases flow for ANC clinic is 520 per month. The K^th^ value was 520/226 which is equal to 2.3, so every 2nd woman was asked. The first comer woman was selected as of a first woman and every second woman was asked.

### 2.4. Data Collection Tools and Techniques

An interviewer-administered questionnaire was developed for the purpose of data collection after reviewing the relevant literature. It was prepared originally in English and translated to the local language, Amharic, for the purpose of data collection and then it was translated back to English again for consistency. The questionnaire had two parts like sociodemographic and utilization parts. Face to face interview was carried out by two diploma holder midwives under the guidance of one BSc midwife supervisor for a period of one month. The quality of data was ensured through training of data collectors and supervisors for two days, close supervision, and prompt feedback. In addition, a regular manual check-up for completeness and consistency of the data was made on daily basis. Prior to the data collection pretest was made on 5% of the total sample size of the respondent's in Addis Zemen health center.

### 2.5. Operational Definition

 
**Frequently utilization of ITNs: **if a woman uses ITNs in every night.
 
**Frequently checking ITNs for holes:** if a woman checks ITNs for hole at least once a week
 
**Utilization of ITNs** was measured based on six ITNs utilization related questions
  - ** Good practice**: those women who scored 50% and more correct response for ITNs utilization related questions were considered as had good practice [28].
  - ** Poor practice**: those women who scored less than 50% correct response for ITNs utilization related questions were considered as had poor practice [28].

### 2.6. Data Analysis

After checking the data for completeness manually, the collected data were entered into epidata software version 3.1 and finally cleaned and analyzed by using SPSS V-20. Descriptive statics of different variables were presented by frequency and percentage using tables, bar, and pie charts. For descriptive numerical variables mean and the standard deviation were computed. Binary and multivariate logistic regressions were computed to identify factors associated with ITN utilization at 95% confidence interval. Variables with a P value of< 0.25 in binary logistic regression were transferred into multivariate logistic regression. Variables which had P-value <0.05 in multivariate logistic regressions were considered significantly associated with ITN utilization. The odds ratio was used to determine the direction and strength of the association.

### 2.7. Ethical Considerations

Prior to data collection ethical clearance was obtained from an institutional review board of Debre Tabor University. Participants were informed about the purpose of the study and their full right not to be interviewed at all or at any time while the interview is going on. Informed verbal consent from every participant was obtained before conducting the interview. The address and name of the respondents were not included for the sake of confidentiality. The participants' privacy was ensured by interviewing them where there is no flow of people.

## 3. Result

### 3.1. Sociodemographic Characteristics

A total of 226 subjects participated in making a response rate of 100%. The mean age of the participants was 27.5 years with SD ±5.2. The maximum and minimum age were 44 and 18 years, respectively. Most of the participants 216(95.6%) were married and less than half 96(42.5%) of women were housewives. All 226 (100%) of the participants were Amhara in ethnicity and 183(81.0%) were Orthodox Christian in religion. Eighty-eight (38.9%) of respondents had a monthly household income of 50 or less US dollar. Regarding participants' educational status, nearly one-fourth 62(27.4%) of participants were not able to read and write whereas 36(16.0%) of subjects had an educational status of college and above (Table 1).

### 3.2. Means of Communication

Of all a total of 226 participants, 202 (89.4%) of them had mobile, 125(55.3%) of them had television, 67(29.6%) of respondents had the radio whereas 21(9.3%) of participants had no any type of means of communication.

### 3.3. Utilization of ITN

Almost all (99.6%) of participants had their own ITN. Of a total of 226 subjects, 74.3% of participants reported that they had slept under the ITN on the previous night. Twenty-one (9.3%) participants frequently check the ITN for holes and 70.4% of women frequently sleep under ITN. The overall utilization was measured by five ITN utilization questions and dichotomized into poor utilization and good utilization. Accordingly, 160 pregnant women (70.8%) had good ITN utilization, whereas the rest, 66 (29.2%), of participants had poor utilization (Table 2).

### 3.4. Reason for Not Using ITN

Participants who did not utilize ITN were asked for the reason for not using ITN and unsuitable to use was the major reason which was reported by 10.2% participants. Fear of side effects and lack of awareness were another reasons for not using ITN which were reported by 9.7% and 8.4% of participants, respectively (Table 3).

### 3.5. Associated Factors

Ten variables were tested in binary logistic regression to see the association between dependent and dependent variables. Six variables were found to be significantly associated (p=<0. 25) with ITN utilization. In multivariate logistic regression only women's age, educational status, occupation, income, and husband educational status were predictor variables of ITN utilization. Mothers who had an educational status of college and above were 2.8 times more likely utilized ITN than mothers who could not read and write (AOR; 2. 8: CI; 1.9, 6.5). Mothers whose age was greater than 30 were 70% times less likely to utilize ITN than mothers whose age was 30 and less (AOR; 0.3: CI;.2,0.6). Mothers whose husband educational status was college and above were 1.7 times more likely to utilize ITN than mothers whose husband could not read and write (AOR; 1.7, CI; 1.5,6.5) (Table 4).

## 4. Discussion

The finding of this study revealed that the overall utilization of ITN by pregnant women is 70.8%. This result is similar to a study done in Nigeria (71.5%) [32]. This finding is a little bit comparable with studies done in Uganda (66.8%) [38] and Ghana (66.1%) [21]. However, the result of this research is higher than studies done in Nigeria in 2012(39.1%), 2014(49.2%), and 2017 (49.6%) [22, 25, 26]. The possible explanation of the difference may due to time variation, sociodemographic difference, and study design difference. The other possible explanation may be due to the free distribution of ITN by governmental and nongovernmental organizations in Ethiopia. This finding is also higher than studies done in Uganda (35%) [39] and Kenya (13%) [29]. High utilization of ITN by pregnant women in the current study may be due to high (96.6%) awareness of ITN by pregnant women when compared to the previous studies. And this difference may be explained by time variation and study design difference.

Similarly, the finding of this study is higher than studies done in Ethiopia in different areas like Raya Zebo (58.1%) [27] and Shashongo (15.4%) [28]. This difference may be due to the time difference between the studies which means there is an average of 7-year difference and with this time variation different strategies were implemented. And also another possible explanation of the difference may be due to the fact that there was a study design difference, in previous studies, and the study design was community-based study design which includes women had no ANC follow-up. In contrary the finding of this study is lower than a study done in Rwanda (84.5%) [30]. This difference may be due to the fact that there was a large sample size (13,671) in the previous study. Similarly, this figure is slightly lower than a study done in Congo (78.4%) [20]. Low utilization of ITN in the current study may be due to the fact that there are high proportions of women with a lower level of education.

This study assessed predictor variables of ITN utilization by pregnant women. In multivariate logistic regression only women's age, educational status, income, and husband educational status were found to be predictor variables of ITN utilization. Women whose age was greater than 30 years old were 70% less likely utilized ITN than women who were 30 or less years old (AOR; 0.3: CI; 0.2, 0.6). The finding of this study is similar to studies done in sub-Saharan Africa [33], Sudan [34], and Ethiopia [28]. The association may be due to the fact that younger women may understand information easily and seek health care. However, this result is not in line with a study done in Nigeria in which older women more utilized ITN than elder women.

This study also revealed that the utilization of ITN was influenced by respondents' educational status. Respondents who had an educational status of college and above were 2.8 times more likely utilized ITN than respondents who could not read and write (AOR; 2.78, CI; 1.9,6.1). Similarly, studies done in Cameroon [35], Nigeria [32], Sub-Saharan Africa [33], and Uganda [36] revealed that respondents who had higher educational status more likely utilized ITN than respondents who had no formal education. This finding also agrees with studies done in Ethiopia [27, 28]. The association may be due to that fact that educated mothers can easily read and understand the information regarding malaria and ITN. Additionally, educated mothers may seek health care and may refer to newsletters, magazines, and books.

In this study, household monthly income was significantly associated with utilization of ITN by pregnant women. Participants who had the household monthly income of 151 US dollars or more were 2.2 times more likely utilized ITN than mothers who had the household monthly income of 50 US dollars or less (AOR; 2.2: CI; 1.7, 5.4). This finding is supported by studies done in Cameroon [35], Sudan [34] and Nigeria [32, 37]. The result of this study also in line with studies done in sub-Saharan Africa and Ethiopia [12, 27, 28]. This association may be due to the fact that mothers who have better income may be more likely exposed to health institutions and may get information regarding malaria and ITN.

In our study, the husband educational status was found to be significantly associated with ITN utilization. Mothers whose husband educational status was college and above were 1.7 times more likely utilized ITN than mothers whose husband could not read and write (AOR; 1.7, CI; 1.5,6.5). However; we could not find any article that shows the association between husband educational status and ITN utilization. This association may be due to the fact that educated husbands may explore information regarding malaria and ITN and may share for their spouse. In addition to this, educated husbands may encourage their spouse to use ITN. Different literatures indicate that husband educational status has a great influence on the utilization of maternal and child health service care, wife's mortality, family size, and other health indicators. Upon this, an educational status of pregnant women's husband may influence women to use ITN [40–43].

As the study was a hospital-based cross-sectional study design, the result might not be a true representative of the community. As one of the limitations of quantitative research is not addressing participants' feeling, our study also shared this limitation. Another limitation of this study was it shared the limitation of using small sample size since it was conducted on small sample size.

## 5. Conclusion

Utilization of insecticide bed net by pregnant women is low. This finding confirmed that mothers' age, educational status, income, and husband educational status were predictor variables of ITN utilization. The finding of this study concluded that having a high educational status, having better income, having a husband who has higher educational status, and being elder lead to utilize ITN. Different stakeholders shall give a special attention to awareness creation on advantageous of insecticide bed net.

## Figures and Tables

**Table 1:** Sociodemographic characteristic of respondents in Addis zemen primary hospital, northwestern, of Ethiopia 2018 (n=226).

**Variable **	**Category**	**Frequency**	**Percent (**%**)**
Age	15-24	67	29.7
	25-34	132	58.4
	35-44	27	11.9
	Total	226	100

**Residence **	Urban	155	68.5
	Rural	71	31.5
	Total	226	100

**Marital status**	Married	216	95.6
	Divorced	1	0.4
	Separated	4	1.8
	Cohabited	5	2.2
	Total	226	100

**Religion**	Orthodox	183	81.0
	Muslim	35	15.5
	Protestant	8	3.5
	Total	226	100

**Occupation**	Housewife	96	42.5
	Governmental employee	34	15
	Merchant	95	42.1
	Daily labor	1	0.4
	Total	226	100

**Education**	unable to read and write	62	27.4
	able to read and write	31	13.7
	primary education (1-8)	66	29.2
	secondary education (9-12)	31	13.7
	college or university	36	16
	Total	226	100

**Monthly income**	50 or less$	88	38.9
	51-100$	47	20.8
	101-150$	61	27
	151 or more$	30	13.3
	Total	226	100

**Husband educational status **	unable to read and write	57	25.2
	able to read and write	36	16
	primary education (1-8)	63	27.9
	secondary education (9-12)	22	9.7
	college or university	48	21.2
	Total	226	100

**Husband occupation**	Farmer	73	32.3
	government employee	52	23
	Merchant	101	44.7
	Total	226	100

**Table 2:** ITN utilization by pregnant woman in Addis zemen primary hospital, northwestern, of Ethiopia 2018 (n=226).

**Variable **	**Category **	**Frequency**	**Percent (**%**)**
Own ITN	Yes	225	99.6
	No	1	0.4
	Total	226	100

**Re treat ITN**	No	226	100
	Total	226	100

**Cheek ITN hole**	Yes	21	9.3
	No	205	90.7

**Sleeping under ITN frequently**	Yes	159	70.4
	No	67	29.7
	Total	226	100

**Sleep under ITN previous day**	Yes	168	74.3
	No	58	25.7
	Total	226	100

**Table 3:** Listed reasons for not using ITN by the pregnant woman in Addis Zemen primary hospital, northwestern, of Ethiopia, 2018 (n=226).

**Reason for not using ITN (n=64)**	Frequency	Percentage
Lack of awareness	19	29.7
fear of side effects	22	34.4
Unsuitable to use.	23	35.9
Total	64	100

**Table 4:** Factors associated with ITN utilization in Adis Zemen Hospital, Northwestern Ethiopia, 2018 (n=226).

**Variables **	**ITN Utilization**		**Crude Odd Ratio (95**%**CI)**	**Adjusted Odd Ratio (95**%**CI)**
	**Yes**	**No**		
**Age**				
≤30	128	46	1	
>30	32	20	.6(.3-1.0)	.3(.2-.6) *∗∗*
Residence				
Urban	109	46	1	
Rural	51	20	1.3(0.6-4.5)	
**Religion **				
Orthodox	129	54	1	
Others	31	12	1.1(.5-2.3)	
**Marital status**				
Married	153	63	1	
Others	7	3	1.1(.3-4.2)	
**Educational status**				
Unable to read and write	41	21	1	1
Able to read and write	17	14	.6(.3-1.5)	.5 (0.3-1.3)
Primary education	46	20	1.2(.6-2.5) *∗*	1.5 (0.8-2.1)
Secondary education	24	7	1.8(.7-4.7) *∗*	1.9 (.5-1.8)
College and above	32	4	4.1 (2.8-7.6)*∗∗*	2. 8(1.9-6.5)*∗∗*
**Occupation **				
Housewife	67	29	1	1
Governmental employee	31	4	3.4 (1.8-7.7)*∗∗*	1.6 (0.8-.4-1)
Market trade vendor	62	33	.8(.4-1.5)	.6(.2-3.3)
**Income **				
50 or less$	55	33	1	1
51-100$	34	13	1.6(.7-3.4)	2.0(.3 -7.8)
101-150$	46	15	1.8(.9-3.8) *∗*	1.7(.6-4.1)
151 or more$	25	5	3 (1.1-6.6)*∗∗*	2.2(1.7-5.4)*∗∗*
**Husband occupation**				
Farmer	47	26	1	1
Governmental employee	44	8	3.1(1.3-7.1)*∗∗*	1.8 (.2-10.5)
Market trade vendor	69	32	1. 2(.6-2.6)	1.2 (.3 -5.3)
**Husband Educational status**				
Unable to read and write	37	20	1	
Able to read and write	21	15	.8(.3-1.8)	.8(.3.2.7)
Primary education	47	16	1.6(.7-3.5) *∗*	1.2(.3-5)
Secondary education	14	8	.9(.3-2.6)	.5(.07-2.8)
College and above	41	7	3.2(2.2-8.3) *∗∗*	1.7(1.5-6.1) *∗∗*
**Communication **				
Yes	148	57	1	
No	12	9	.5(.2-1.3) *∗*	.3(.6-3.6)

***Note.*** *∗∗* indicates p-value<0.05 and CI=confidence interval*∗* indicates p-value<0.25.

## Data Availability

The data used to support the findings of this study are available from the corresponding author upon request.

## List of Abbreviations

ANC: Antenatal care
AOR: Adjusted odds ratio
CI: Confidence interval
ITN: Insecticide bed net
SD: Standard deviation
USA: United States of America.

## Additional Points

Insecticide-treated bed net (ITN) is a type of vector control approach which has to be treated with chemicals and it is a cost-effective type of malaria prevention. Malaria during pregnancy has different impacts like maternal infection, maternal anemia, low birth weight, prematurity, and others. Malaria infection during pregnancy is a major health problem in sub-Saharan Africa including Ethiopia. So, assessing utilization and associated factors of ITN is very important. This hospital-based cross-sectional study was conducted in Adis Zemen primary Hospital. After obtaining informed consent, data were collected using a pretested structured questionnaire via face to face interview for a period of one month. A total of 226 sampled pregnant mothers participated and systematic random sampling technique was used to reach the study unit. The participants' mean age was 27.5 years with ±5.2 SD years. The finding of this study revealed that all 226 (100%) of the participants were Amhara in ethnicity and 183 (81.0%) were Orthodox Christian in religion. This finding revealed that the ITN utilization by pregnant women is 70.8%.Utilization of ITN by pregnant women is low in Adis Zemen. This finding confirmed that mothers' age, educational status, income, occupation, and husband educational status were predictor variables of ITN utilization. The finding of this study suggests that there is a need to give emphasis and deliver health education about ITN.
